# Surgical resection of hepatic metastases from colorectal cancer: A systematic review of published studies

**DOI:** 10.1038/sj.bjc.6603033

**Published:** 2006-03-14

**Authors:** P C Simmonds, J N Primrose, J L Colquitt, O J Garden, G J Poston, M Rees

**Affiliations:** 1 Cancer Research UK Clinical Centre, MP824, Southampton General Hospital, Tremona Road, Southampton SO16 6YD, UK; 2 University Surgery, F Level Centre Block (MP816), Southampton General Hospital, Southampton SO16 6YD, UK; 3 Department of Clinical and Surgical Sciences, Royal Infirmary of Edinburgh, 51 Little France Crescent, Old Dalkeith Road, Edinburgh, Scotland EH16 4SA, UK; 4 Department of Surgery, University Hospital Aintree, Longmoor Lane, Liverpool L9 7AL, UK; 5 Hepatobiliary Surgery Unit, North Hampshire Hospital, Aldermaston Road, Basingstoke RG24 9NA, UK

**Keywords:** colorectal liver metastases, surgery, systematic review, survival, morbidity

## Abstract

No consensus on the indications for surgical resection of colorectal liver metastases exists. This systematic review has been undertaken to assess the published evidence for its efficacy and safety and to identify prognostic factors. Studies were identified by computerised and hand searches of the literature, scanning references and contacting investigators. The outcome measures were overall survival, disease-free survival, postoperative morbidity and mortality, quality of life and cost effectiveness, and a qualitative summary of the trends across all studies was produced. Only 30 of 529 independent studies met all the eligibility criteria for the review, and data on 30-day mortality and morbidity only were included from a further nine studies. The best available evidence came from prospective case series, but only two studies reported outcomes for all patients undergoing surgery. The remainder reported outcomes for selected groups of patients: those undergoing hepatic resection or those undergoing curative resection. Postoperative mortality rates were generally low (median 2.8%). The majority of studies described only serious postoperative morbidity, the most common being bile leak and associated perihepatic abscess. Approximately 30% of patients remained alive 5 years after resection and around two-thirds of these are disease free. The quality of the majority of published papers was poor and ascertaining the benefits of surgical resection of colorectal hepatic metastases is difficult in the absence of randomised trials. However, it is clear that there is group of patients with liver metastases who may become long-term disease- free survivors following hepatic resection. Such survival is rare in apparently comparable patients who do not have surgical treatment. Further work is needed to more accurately define this group of patients and to determine whether the addition of adjuvant treatments results in improved survival.

Colorectal cancer is the third most common cause of cancer death in the UK (Cancer Research UK Information Resource Centre, 2003). Surgery is the treatment of choice for patients with localised disease but over half of all patients will develop metastases. The liver is often the first site of metastatic disease and may be the only site of spread in as many as 30–40% of patients with advanced disease (Weiss *et al*, 1986; Hugh *et al*, 1997a).

It has been postulated that because haematogenous spread usually occurs in a stepwise fashion, initially to the liver, with subsequent intrahepatic spread via the portal vein and further spread to the systemic circulation, surgical resection of isolated hepatic metastases from colorectal cancer may be curative.

The natural history of metastatic colorectal cancer is variable, with a median survival without treatment of only 8 months (Seymour *et al*, 1997; Simmonds, 2000). Patients with isolated hepatic metastases have a better prognosis than those with more extensive metastatic disease (Goslin *et al*, 1982; Lahr *et al*, 1983; Stangl *et al*, 1994; Rougier *et al*, 1995) suggesting biological differences in the two settings (Goslin *et al*, 1982; Lahr *et al*, 1983; Stangl *et al*, 1994; Rougier *et al*, 1995). However, few patients with limited liver-only metastases survive for 5 years (Goslin *et al*, 1982; Stangl *et al*, 1994).

A variety of therapeutic approaches have been used in the treatment of metastatic colorectal cancer including surgery, chemotherapy, radiofrequency ablation, cryotherapy or some combination of these (Primrose, 2002). Around 20–30% of patients with liver-only metastases are potentially resectable (Nordlinger *et al*, 1994; Stangl *et al*, 1994; Jeffery *et al*, 2002). The selection criteria for surgery are usually controlled primary tumour, no extra hepatic metastases and that resection is technically feasible with tumour-free margins (Hugh *et al*, 1997b). A small proportion of patients with completely resectable extrahepatic disease may become long-term survivors (Scheele *et al*, 1995). Chemotherapy used alone is palliative but may prolong the survival of patients with unresectable disease (de Gramont *et al*, 2000; Douillard *et al*, 2000; Saltz *et al*, 2000; Simmonds, 2000). Used in combination with surgery it may prolong the time to recurrence after resection of hepatic metastases (Kemeny *et al*, 2002) or downsize to resectability patients previously judged inoperable (Giacchetti *et al*, 1999). The role of other treatments is poorly defined (Armstrong *et al*, 1994; Donovan, 1995; Adam *et al*, 1997; de Baere *et al*, 1999; Primrose, 2002).

Although there are a large number of published studies reporting the results of surgical resection of hepatic metastases from colorectal cancer, the effectiveness and cost effectiveness of such surgery remains unclear. This systematic review has been undertaken to assess the published evidence for the efficacy and safety of this intervention and, by examining potential prognostic factors, identify patients who may either benefit from surgery or in whom such intervention is inappropriate.

## METHODS

### Criteria for selecting studies

As randomised trials comparing surgical resection *vs* no surgery have never been conducted, we have attempted to identify all relevant prospective and retrospective series reporting the outcomes of surgical resection with curative intent of colorectal hepatic metastases. Patients undergoing repeat hepatic resection were also included. In order to ensure that the surgical series reviewed reflected the outcomes for patients treated with modern surgical, anaesthetic and supportive care techniques, we have restricted our qualitative analysis to surgical series published after 1980 reporting the outcomes of at least 100 patients. Studies were required to follow patients for at least 30 days for inclusion of data on postoperative morbidity and mortality, and for a median of at least 24 months for inclusion of survival data. No language restriction was applied on searching. All eligible European language studies were translated, but retrospective non-European articles were not.

The outcome measures were overall survival (1 year/3 year/5 year), disease-free survival (1 year/3 year/5 year), operative and postoperative morbidity, operative and postoperative mortality, quality of life and cost effectiveness.

### Search strategy

We searched the following electronic databases: Cochrane Controlled Trials Register, Medline, Embase, Cancerlit, Science Citation Index, Edina Biosis, CINAHL and NHS Economic Evaluation Database. Both medical subject heading and free text searching were used to improve the sensitivity of the search. The search strategy was piloted and modified to improve the hit rate, and the sensitivity of the search strategy was tested by hand searching three journals in which a high proportion of identified studies had been published (*Cancer*, *British Journal of Surgery*, and *Diseases of the Colon and Rectum*, 1998–1999). All relevant studies had been identified, confirming that the search was comprehensive. Searching took place between May 2000 and October 2000. The full search strategy is available from the authors on request.

The following registers were searched for details of ongoing and unpublished studies: National Research Register, Current Controlled Trials, MRC Funded Research database, UKCCCR trials register, Centre Watch Clinical Trials listing, Physician Data Query (USA), National Institutes of Health Clinical Trials (USA), National Health and Medical Research Council (Australia), Trial Amnesty on the Cochrane library, System for Information on Grey Literature in Europe (SIGLE). Investigators of eligible prospective studies and large surgical centres were contacted to find further published, unpublished or ongoing studies, and the reference lists of review articles on this subject and all eligible studies were examined. In order to identify studies that had not been indexed in the above sources, the Proceedings of the American Society of Clinical Oncology (1982–2000) were hand searched.

### Review procedures and analysis

All relevant studies were assessed according to the above inclusion criteria using a standard checklist performed independently by two reviewers (one clinical and one non-clinical) and agreement on eligibility was reached. Investigators were contacted for further information where eligibility could not be determined from the published study. Data were extracted from published papers by one reviewer using a standard data extraction sheet and then verified independently by a second reviewer. Data extracted included demographic characteristics of patients, preoperative comorbidities, details of the primary tumour, details of hepatic and extrahepatic metastases, type of resection, nonsurgical therapy and outcomes. When a study had generated multiple publications, the most recent was used to extract data on relevant outcome variables. Earlier publications were used to provide information on baseline characteristics or methodology where necessary. Validity was assessed independently by two reviewers using a standard critical appraisal checklist. For the purposes of this review, items relevant to the assessment of uncontrolled surgical case series were combined from two checklists (Cowley, 1995; Downs and Black, 1998). The checklist was piloted by two reviewers and assessed quality of reporting and internal and external validity. An index of inter-rater agreement was calculated using the Kappa statistic. As all of the eligible published studies were either prospective or retrospective case series, a qualitative summary was produced to identify and describe trends across all studies.

## RESULTS

### Description of studies identified

Of 529 studies identified by our initial search strategy, 470 were not included in the review, often for more than one reason: 409 (77%) reported less than 100 patients, the median follow-up was less than 24 months in 45 (9%) and unclear in 345 (65%) studies. Other reasons for exclusion were combined data from colorectal and non-colorectal liver metastases (38/529) and lack of mortality and survival data (21/529). The eligibility of 19 publications was unclear, but as these were updated elsewhere the older reports were excluded, and 15 studies were not included because their eligibility remained unclear despite seeking further details from the authors of the original report. A list of excluded studies with reasons for exclusion is available on request from the authors.

Forty-nine published and unpublished studies met all the inclusion criteria, including multiple publications presenting updated information or data on different aspects of the same patient population, providing 30 independent eligible studies. Eighteen of these were single publications (Savage and Malt, 1992; Sugihara *et al*, 1993; Donato *et al*, 1994; Doci *et al*, 1995; Fernandez-Trigo *et al*, 1995; Fuhrman *et al*, 1995; Beckurts *et al*, 1997; Rees *et al*, 1997; Cady *et al*, 1998; Ohlsson *et al*, 1998; Riesener *et al*, 1998; Bradley *et al*, 1999; Harmon *et al*, 1999; Harms *et al*, 1999; Kemeny *et al*, 1999a; Bolton and Fuhrman, 2000; Okano *et al*, 2000; Moroz *et al*, 2002) and in a further 12 cases the data was obtained from multiple publications (Hughes *et al*, 1988, 1989; Hohenberger *et al*, 1990, 1994; Scheele *et al*, 1990, 1991, 1995, 1996; Steele Jr *et al*, 1991, 1995; Rosen *et al*, 1992; Gayowski *et al*, 1994; Stangl *et al*, 1994; Fong *et al*, 1995, 1997, 1999; Leslie *et al*, 1995; Jamison *et al*, 1996, 1997; Jenkins *et al*, 1997; Bakalakos *et al*, 1998a, 1998b; Iwatsuki *et al*, 1999; Minagawa *et al*, 1999; Ambiru *et al*, 1999a; Kemeny *et al*, 1999b, 1999c; Brand *et al*, 2000; DeMatteo *et al*, 2000; Minagawa *et al*, 2000). In addition, data on 30-day mortality and morbidity were included from a further nine studies (one with two publications) in which survival data was excluded owing to insufficient or unclear follow up (Minton and Abou-Issa, 1990; Fegiz *et al*, 1991; Nordlinger *et al*, 1992; van Ooijen *et al*, 1992; Wade *et al*, 1996; Lorenz *et al*, 1997, 1998; Nadig *et al*, 1997; Taylor *et al*, 1997; Figueras *et al*, 2001). Details of all studies are tabulated in Appendix A.

Just two studies included all patients presenting with colorectal liver metastases (Scheele *et al*, 1990, 1991, 1995, 1996; Stangl *et al*, 1994; Harms *et al*, 1999) and only three studies presented data on all patients undergoing surgery (radical, nonradical and laparotomy) (Steele Jr *et al*, 1991, 1995; Fuhrman *et al*, 1995; Bakalakos *et al*, 1998a). The majority (27) of studies presented data on resected patients, but while some studies distinguished between R0 resections (no residual disease, histologically clear margins), R1 (histologically involved margins) and R2 resections (macroscopic residual disease), in many this was unclear. Six studies included only a select group of patients undergoing radical resection, and one study included patients undergoing repeat resection only.

Eleven studies were conducted prospectively, three of which were randomised controlled trials comparing surgery alone with surgery plus chemotherapy (Lorenz *et al*, 1998; Kemeny *et al*, 1999a) or comparing two adjuvant chemotherapy regimes (Kemeny *et al*, 1999c). Twenty-five studies were retrospective and the design of three studies was unclear. Twenty-six studies were conducted in single centres, 11 were multicentre studies and two were undertaken by two centres.

No data was found on comorbidity, quality of life, or cost effectiveness of liver resection.

### Methodological quality of included studies

There was consistency in validity appraisal between the two reviewers, as indicated by a Kappa statistic of 0.79. The 16-point validity scale was completed for all studies, including one study that was published in abstract form only (Kemeny *et al*, 1999a) (Appendix B). Total scores ranged from 2 to 14 (mean 8.7, median 9).

The quality of reporting was poor in many studies. Eligibility criteria for surgery or inclusion criteria in the study were not reported by 12 (31%) studies. The distribution of principal confounders, such as synchronous or metachronous metastases and curative intent of surgery were not reported by almost a third of studies. Basic demographic information such as mean age and sex distribution was also often missing. Patients were thought to be representative in only a minority of studies (mostly highly selected), and similarly the treatment centres were considered to be unrepresentative (highly specialised).

The primary end points of mortality and survival were not clearly defined in 33 and 74% of studies, respectively; therefore, the comparability of results between studies is unclear. Postoperative mortality was excluded from survival analysis in 21% studies, and patients lost to follow-up were not reported or not included in the appropriate analysis by over half of the studies. Median length of follow-up was not reported by 31% of studies. The outcome of patients receiving surgery alone was reported by just 12 (31%) studies. In 10 (26%) studies patients receiving additional therapies such as adjuvant chemotherapy were not analysed separately. Further, the use of additional therapies was not stated in 17 (44%) studies.

### Postoperative mortality

Death within 30 days of hepatic resection was reported by 24 studies, ranging from 0 to 6.6% of patients (median 2.8%). A further nine studies reported perioperative mortality within an undefined time period (1.3–4.6%, median 3.6%), and two studies reported 60-day mortality (3.4–5.5%). Mortality was not reported by four studies. Cause of death was reported by 15 studies for a total of 103 patients (Table 1), including two studies that reported deaths from surgical complications occurring within 90 days (Iwatsuki *et al*, 1999) and 128 days (Scheele *et al*, 1996).

### Postoperative morbidity

Perioperative complications, including indicators of morbidity such as length of hospital stay, were reported by 29 studies. However, some studies reported fatal complications only (van Ooijen *et al*, 1992; Scheele *et al*, 1996; Lorenz *et al*, 1998; Bolton and Fuhrman, 2000; Moroz *et al*, 2002), length of hospital stay only (Hohenberger *et al*, 1994; Fuhrman *et al*, 1995), blood transfusions only (Jamison *et al*, 1997) or simply reported the number of patients with nonspecified complications (van Ooijen *et al*, 1992; Fernandez-Trigo *et al*, 1995; Scheele *et al*, 1996; Jenkins *et al*, 1997; Taylor *et al*, 1997; Bakalakos *et al*, 1998a; Fong *et al*, 1999; Figueras *et al*, 2001).

The number of studies reporting each complication and the number of patients with the complication is presented in Table 2.

### Overall survival

Two studies reported overall survival at 5 years for all patients undergoing surgery (resection and laparotomy only), median 23% (15–31%) (Table 3). Studies in which it was unclear whether resections were R0 or R1/2, or only presented data for both types of resection combined, had a median 5-year survival of 32% (9–63%). Sixteen studies presented 5-year survival for patients undergoing R0 resection, either for the whole study population or for subgroups of patients. Median 5-year survival for these studies was 30% (range 15–67%). Eleven studies reporting 5-year survival for nonradical resections had a median 5-year survival of 7.2% (range 0–30%), and six studies reporting patients who did not undergo resection had a median 5-year survival of 0% (range 0–6%). Survival according to study design is also presented in Table 3. Patients who underwent resection without any additional therapy had a median 5-year survival of 30% (range 20–33.6%), whereas patients who received additional therapy had a median 5-year survival of 35.5% (range 9–63%). Studies in which use of additional therapy was not stated reported 5-year survival between 14 and 58% (median 32.5%). Overall 5-year survival was not reported by three studies (Donato *et al*, 1994; Hohenberger *et al*, 1994; Cady *et al*, 1998), and one study reporting survival from repeat hepatic resections only was not included in this analysis (Fernandez-Trigo *et al*, 1995).

### Disease-free survival

Disease-free survival was reported by fewer studies. Median disease-free survival was 11.6 (Hohenberger *et al*, 1994) and 17 (Doci *et al*, 1995) months (median 14.3 months) for radically resected patients and between 10.8 and 37.4 months (median 17.2 months) for patients with unspecified resections (Jenkins *et al*, 1997; Ohlsson *et al*, 1998; Kemeny *et al*, 1999c; Minagawa *et al*, 2000).

Five-year disease-free survival was reported between 9 and 35% (median 18%) (Doci *et al*, 1995; Scheele *et al*, 1996; Ambiru *et al*, 1999b) and 4–47% (median 21%) (Hughes *et al*, 1989; Jenkins *et al*, 1997; Ohlsson *et al*, 1998; Bradley *et al*, 1999; Iwatsuki *et al*, 1999; Minagawa *et al*, 2000; Okano *et al*, 2000) by studies with radically resected patients and unspecified resections, respectively.

### Sites of recurrence

Sites of recurrence following hepatic resection were reported by 13 studies (Table 4). They were classified as liver, extrahepatic or liver plus extrahepatic. Twenty-two percent of all patients experienced recurrence in the liver only, although this is likely to be underestimated as two studies did not specify the proportion of liver-only recurrences. Liver plus extrahepatic recurrences and extrahepatic-only recurrences were experienced by 16 and 24% of patients, respectively. In addition, one study reported recurrences in 235 (62.5%) radically resected patients, although sites of recurrence were not specified.

### Prognostic factors

Many studies reported 1-year, 3-year, 5-year and median overall survival and disease-free survival for various potential prognostic factors relating to the characteristics of the primary tumour and/or liver metastases. In general, comparison between studies is limited owing to differences in categorisation of the prognostic factors. A definition of ‘synchronous’ is not given in most cases, but could mean hepatic resection within 1 month (Hughes *et al*, 1989), 3 months (Jamison *et al*, 1997; Rees *et al*, 1997) or 6 months (Donato *et al*, 1994) of primary surgery. Time interval between resection of the primary tumour and metastases has been classified into five different categories. Categories for number and size of liver metastases are also extremely varied, and reporting of the type of liver resection is inconsistent. Results from multivariate analysis, which identified prognostic factors independently associated with overall survival, are shown in Table 5. As the factors included in the model differed for each study these cannot be directly compared.

### Repeat resections

In studies that reported this outcome, between 3.6 and 17% (median 9%) of patients underwent repeat hepatic resection (Scheele *et al*, 1996; Rees *et al*, 1997; Ohlsson *et al*, 1998; Bakalakos *et al*, 1998a; Bradley *et al*, 1999; Iwatsuki *et al*, 1999; Ambiru *et al*, 1999b; Kemeny *et al*, 1999c; Minagawa *et al*, 2000). Three hepatic resections were performed on 0.9–4% (median 3.8%) of patients (Ohlsson *et al*, 1998; Bakalakos *et al*, 1998a; Minagawa *et al*, 2000), and just 0.4% underwent four resections (Minagawa *et al*, 2000). The Repeat Hepatic Registry reported a series of 170 patients undergoing repeat resection, of which 4.7% underwent a third resection (Fernandez-Trigo *et al*, 1995). Median and 5-year overall survival from the date of second liver resection for the 170 patients was 34 months and 32%, respectively.

## DISCUSSION

Although the prognosis of metastatic colorectal cancer is poor with few patients surviving for 5 years or more (Stangl *et al*, 1994) long-term survival has been reported following surgical resection of isolated hepatic metastases (Fong and Salo, 1999; Geoghegan and Scheele, 1999). The nature of the published studies of surgical resection for colorectal liver metastases did not allow for a quantitative analysis to be performed and we have thus undertaken a qualitative systematic review to summarize the available evidence for the effectiveness of this intervention.

Surgical resection of hepatic metastases from colorectal cancer can be undertaken safely in the majority of patients. The median postoperative (30 day) mortality reported by 24 studies was only 2.8% (0–6.6%). There was patchy reporting of the causes of postoperative death, with the most frequent causes being hepatic failure, postoperative haemorrhage, and sepsis. It is likely that current surgical and anaesthetic practice is associated with perioperative mortalities nearer to 1% as demonstrated in a recent multicentre trial (Nordlinger *et al*, 2005). Operative morbidity was more difficult to quantify as many studies reported only fatal morbidity or a very limited range of postoperative complications. It was therefore difficult to determine the proportion of patients experiencing operative morbidity and thus its overall impact on patients in the majority of studies. Some studies presented information on outcomes that may be surrogate markers for operative morbidity. Two studies reported information on the duration of stay in intensive care following surgery, the median time in one study was 1 day, while the mean from the two studies was 3 days. These limited results suggest that most patients require only a short period of intensive care following hepatic resection for colorectal metastases. The median or mean length of hospital stay reported by 17 studies ranged from 7–21 days indicating that while most patients made a rapid recovery following surgery, some experienced a more prolonged hospital stay possibly as a result of complications from their surgery. None of the studies included in this review presented information on recovery of patients' functional status or quality of life following discharge from hospital.

Two studies presented outcome data for all patients with isolated colorectal hepatic metastases who underwent surgery, resection and laparotomy only, with 15 and 31% of patients surviving 5 years, respectively (Fuhrman *et al*, 1995; Harms *et al*, 1999). Studies including only patients who underwent resection reported a survival of around 30% at 5 years for patients undergoing potentially ‘curative’ resection of isolated hepatic metastases and the majority are disease free at this time. The survival of patients undergoing R0 resections were substantially better (32% at 5 years) than for patients undergoing R1 resections (7.2% at 5 years) and those who did not undergo resection (0% at 5 years).

Patients undergoing surgery may have a better prognosis than other patients with metastatic colorectal cancer as their disease is both confined to the liver and circumscribed within it. The patients are also more likely to be of good performance status and have little or no comorbidity. Identifying a comparable control group in the absence of randomised trials is difficult. Goldberg *et al* (1998) identified 548 patients with recurrent colorectal cancer in a longitudinal study, of whom 222 (41%) were thought to be suitable for ‘curative intent’ surgery. Potentially curative surgery was performed in 109 (20%) and of these 28 (5%) were performed for isolated liver metastases. The estimated recurrence-free survival of patients following liver resection was 32% at 5 years, similar to survival after lung resection and complete resection of local recurrence. The 5-year survival after curative surgery at other sites and multiple sites appeared worse (16 and 0%, respectively). Although not stated, the survival of the patients not treated surgically or who had palliative surgery was by implication poor, but two out of 19 patients with circumscribed disease liver or lung disease treated nonsurgically were alive at final follow-up. A small number of retrospective studies have attempted to determine the natural history of patients with isolated liver metastases. In a review of 484 untreated patients with liver metastases from colorectal cancer, those with the best prognosis (⩽25% liver involvement, primary tumour grade 1/2, no extra hepatic tumour and no mesenteric nodal involvement), had a median survival of 21.3 months, compared with 30 months in patients undergoing hepatic resection in the same institution (Stangl *et al*, 1994). In a group of 125 patients with liver-only metastases, most of whom had had no therapy, the median survival was 12.5 months. All patients died within 5 years. Survival correlated with the extent of liver disease. The presence of three or fewer liver metastases was associated with a median survival of 24 months (Goslin *et al*, 1982). Lahr *et al* (1983) studied 175 untreated patients with liver metastases from colorectal cancer. The median survival of these patients was 6.1 months and the longest survivor lived for 67 months. Patients with 1–4 liver metastases lived longer than those with five or more metastases (median survival 11.8 *vs* 4 months) (Lahr *et al*, 1983). A study of 113 patients with hepatic metastases from colon cancer, reported a mean survival of 3 months in patients with widespread liver disease (Wood *et al*, 1976). Patients with metastases localised to a segment or lobe had a mean survival of 17 months, compared with 25 months for patients with a solitary liver metastases. The overall 1-year survival rates were 6, 27, and 60%, respectively. Another study attempted to distinguish potentially resectable from unresectable disease (Wagner *et al*, 1984). Three groups of untreated patients were studied. Patients with solitary (*n*=39), multiple unilateral (*n*=31) and widespread (*n*=182) had 3-year survivals of 21, 6 and 4%, respectively. However by 5 years virtually all were dead (3, 0 and 2% survival, respectively). Some small studies have examined the outcome of patients with unresected synchronous hepatic metastases (Bengtsson *et al*, 1981; Boey *et al*, 1981; Finan *et al*, 1985; Gorog *et al*, 1997). The median survival of patients in these series ranged from 4.5 to 10.3 months. These studies suggest that there is a small select group of patients with isolated liver metastases from colorectal cancer who may live a long time without surgical intervention. However, their prognosis remains poor because of the inexorable hepatic progression and extrahepatic spread.

Disease recurrence is common after resection of colorectal hepatic metastases indicating that in the majority of cases the extent of the metastatic disease is underestimated by pre and intraoperative staging investigations. Around one-third of patients experienced disease recurrence in the liver alone and may be candidates for repeat resection. The remainder experienced recurrence concurrently in the liver and extrahepatic sites or in extrahepatic sites only. It may be that biological features in the tumour itself may be important as optimal imaging and this requires further study.

Identification of prognostic factors that predict the outcome following surgical resection of colorectal hepatic metastases would assist in the identification of those patients most likely to benefit from this intervention, or more importantly assist in the identification of patients who were unlikely to benefit. Comparison of the different studies included in this review was hampered by differing definitions of the prognostic factors considered for univariate or multivariate analyses. However, some potential prognostic factors were found to be significant in more than half of the studies. We are currently attempting to evaluate these potential prognostic factors using a large prospectively collected data set contributed by multiple centres. This will be published in due course.

This review was performed deliberately on series published up to the millennium as in recent years major changes have occurred in the management of colorectal liver metastases. These factors will almost certainly impact increasingly on the reported outcomes after 2000 (Poston *et al*, 2005). Firstly modern chemotherapy using cytotoxic agents alone offers extension of median survival to 2 years in patients with nonresectable disease (Cals *et al*, 2004; Goldberg *et al*, 2004; Grothey *et al*, 2004; Tournigand *et al*, 2004). When monoclonal biological agents are added to cytotoxic chemotherapy, the prospect of median survival now extends beyond 2 years, and 20% of patients will still be alive 4 years after detection of unresectable liver disease (Cunningham *et al*, 2004; Hurwitz *et al*, 2004; Saltz *et al*, 2004). It is therefore inevitable that the combination of surgical resection and chemotherapy, which is becoming commonplace, will impact on the survival in the surgical series. The EORTC EPOC trial which is the first to randomise liver resection patients to receive additional, modern, chemotherapy is due for reporting in 2007 (Nordlinger *et al*, 2005). Secondly, novel surgical strategies such as preoperative portal vein embolisation to increase residual acceptably safe volume, or two-stage hepatectomy to allow compensatory hepatic hyperplasia before completion of R0 resection (Abdalla *et al*, 2002; Pawlik *et al*, 2005; Poston *et al*, 2005), have also widened the number of resectable patients including those with extensive liver-only disease. These changes in the definition of resectability means that >20% of patients with liver metastases can now be considered for surgery with curative intent at the outset. It is unclear what the long-term outcome of these strategies will be but the results appear encouraging (Abdalla *et al*, 2002; Pawlik *et al*, 2005; Poston *et al*, 2005). This present review should both set the standard for the reporting of subsequent surgical series and provide baseline results to which they may be compared.

In summary there is a substantial body of evidence from prospective and retrospective case series summarised in this review demonstrating that resection of colorectal hepatic metastases can be performed safely with a low mortality rate and around one-third of patients will survive for 5 years or more. These outcomes in highly selected patients exceed those normally associated with metastatic colorectal cancer. Randomised trials comparing surgical resection with nonsurgical treatment are not now possible. Further information must come from well-documented prospective studies examining consecutive series of patients with colorectal cancer and randomised trials comparing liver resection alone with liver resection plus additional chemotherapy.

## Figures and Tables

**Table 1:** Fatal complications

	**No. of studies**	**No. of patients**	**% of reported fatalities (*n*=103)**
Bile leak	4	6	5.8
Perihepatic abscess	2	3	2.9
Hepatic failure	10	19	18.4
Renal failure	4	4	3.9
Generalised sepsis	8	17	16.5
Peritonitis	2	3	2.9
GI bleed	3	3	2.9
Deep vein thrombosis	1	1	1.0
Pulmonary embolism	4	7	6.8
Myocardial infarction	3	3	2.9
Cardiac failure	6	12	11.7
Arrhythmia	1	3	2.9
Pneumonia	2	2	1.9
Cerebrovascular accident	2	3	2.9
Urinary tract infection	1	1	1.0
Postoperative haemorrhage	8	18	17.5
Adult respiratory distress syndrome	2	4	3.9
Multisystem organ failure	2	7	6.8
Anastomotic leak/insufficiency	2	6	5.8
Other	8	14	13.6

NB patients may have had more than one cause of death.

**Table 2:** Morbidity (fatal and non-fatal)

	**No. of studies**	**No. of patients in studies**	**No. of patients with complication**	**% of patients with complication**	
Bile leak	15	3746	148	4.0	
Perihepatic abscess	11	1755	53	3.0	
Hepatic failure	16	3646	102	2.8	
Renal failure	6	643	7	1.2	
Wound infection	10	1618	88	5.4	
Generalised sepsis	12	3312	151	4.6	
GI bleed	6	666	12	1.8	
Deep vein thrombosis	5	897	7	0.8	
Pulmonary embolism	9	935	15	1.6	
Myocardial infarction	5	754	11	1.5	
Arrhythmia	6	777	22	2.8	
Pneumonia	6	933	18	1.9	
Pleural effusion	7	1268	55	4.3	
Urinary tract infection	5	708	15	2.1	
Cerebrovascular accident	5	374	6	1.6	
Peritonitis	2	160	3	1.9	
Cardiac failure	6	532	13	2.4	
Postoperative haemorrhage	15	3913	106	2.7	
Other (including complications not specified)	23	6529	1314	20.1	
					
Any blood transfusion	7	1342	863	64.3	
					
			**Minimum**	**Maximum**	**Median**
*Units transfused*					
Mean	4	572	1	4.4	2.2
Median	6	749	2	6	3
					
*Volume of blood loss (ml)*					
Mean	2	238	989	1900	
Median	2	228	425	1700	
					
*Intensive care (days)*					
Minimum	1	131			0
Maximum	1	131			38
Median	1	134			1
Mean	2	265	2.5	3.5	
					
*Length of stay (days)*	17	2733			
Minimum			1	8	4.5
Maximum			26	100	59
Median			7	17	9.5
Mean			8.8	21	12.5

**Table 3:** Overall 5-year survival

		**5-year survival (%)**		
	**Studies**	**Minimum**	**Maximum**	**Median**
*Patients*				
All patients (resected+non resected)	Fuhrman *et al*, 1995; Harms *et al*, 1999	15	31	
Resected patients (R0/R1 unclear)	Hughes *et al*, 1989; Beckurts *et al*, 1997; Jamison *et al*, 1997; Ohlsson *et al*, 1998; Bakalakos *et al*, 1998a; Bradley *et al*, 1999; Fong *et al*, 1999; Harmon *et al*, 1999; Harms *et al*, 1999; Iwatsuki *et al*, 1999; Kemeny *et al*, 1999a, 1999c; Bolton and Fuhrman, 2000; Minagawa *et al*, 2000; Moroz *et al*, 2002) (Jenkins *et al*, 1997; Okano *et al*, 2000) (Savage and Malt, 1992; Rees *et al*, 1997; Scheele *et al*, 1996	9	63	32
Radical (R0) resection	Savage and Malt, 1992; Sugihara *et al*, 1993; Doci *et al*, 1995; Fuhrman *et al*, 1995; Steele Jr *et al*, 1995; Scheele *et al*, 1996; Beckurts *et al*, 1997; Jenkins *et al*, 1997; Rees *et al*, 1997; Riesener *et al*, 1998; Bakalakos *et al*, 1998a; Fong *et al*, 1999; Harms *et al*, 1999; Iwatsuki *et al*, 1999; Ambiru *et al*, 1999b; Okano *et al*, 2000	15	67	30
Nonradical resection	Lange *et al*, 1989; Savage and Malt, 1992; Sugihara *et al*, 1993; Steele Jr *et al*, 1995; Beckurts *et al*, 1997; Jenkins *et al*, 1997; Rees *et al*, 1997; Bakalakos *et al*, 1998a; Fong *et al*, 1999; Harms *et al*, 1999; Okano *et al*, 2000	0	30	7.2
Patients not resected	Fuhrman *et al*, 1995; Steele Jr *et al*, 1995; Scheele *et al*, 1996; Bakalakos *et al*, 1998a; Harms *et al*, 1999; Moroz *et al*, 2002	0	6	0
				
*Prospective studies*				
Resected (R0/R1 unclear)	Scheele *et al*, 1996; Beckurts *et al*, 1997; Rees *et al*, 1997; Ohlsson *et al*, 1998; Kemeny *et al*, 1999a, 1999c	14	63	32.5
Radical	Steele Jr *et al*, 1995; Scheele *et al*, 1996; Beckurts *et al*, 1997; Rees *et al*, 1997	15	38	30
Non radical	Steele Jr *et al*, 1995; Beckurts *et al*, 1997; Rees *et al*, 1997	0	14	6
				
*Retrospective studies*				
Resected (R0/R1 unclear)	Hughes *et al*, 1989; Savage and Malt, 1992; Jamison *et al*, 1997; Jenkins *et al*, 1997; Bakalakos *et al*, 1998a; Bradley *et al*, 1999; Fong *et al*, 1999; Harmon *et al*, 1999; Iwatsuki *et al*, 1999; Bolton and Fuhrman, 2000; Minagawa *et al*, 2000; Okano *et al*, 2000; Moroz *et al*, 2002	9	58	32.3
Radical	Savage and Malt, 1992; Doci *et al*, 1995; Fuhrman *et al*, 1995; Jenkins *et al*, 1997; Riesener *et al*, 1998; Bakalakos *et al*, 1998a; Fong *et al*, 1999; Harms *et al*, 1999; Iwatsuki *et al*, 1999; Ambiru *et al*, 1999b; Okano *et al*, 2000	17	67	30
Non radical	Savage and Malt, 1992; Jenkins *et al*, 1997; Bakalakos *et al*, 1998a; Fong *et al*, 1999; Harms *et al*, 1999; Iwatsuki *et al*, 1999; Okano *et al*, 2000	0	30	7.2
				
*Single centre*				
Resected (R0/R1 unclear)	Jamison *et al*, 1997; Ohlsson *et al*, 1998; Bakalakos *et al*, 1998a; Harmon *et al*, 1999; Kemeny *et al*, 1999c; Bolton and Fuhrman, 2000; Moroz *et al*, 2002	9	61	32.3
Radical	Savage and Malt, 1992; Sugihara *et al*, 1993; Doci *et al*, 1995; Fuhrman *et al*, 1995; Scheele *et al*, 1996; Jenkins *et al*, 1997; Rees *et al*, 1997; Riesener *et al*, 1998; Bakalakos *et al*, 1998a; Fong *et al*, 1999; Harms *et al*, 1999; Iwatsuki *et al*, 1999; Ambiru *et al*, 1999b; Okano *et al*, 2000	17	67	30
Non radical	Savage and Malt, 1992; Sugihara *et al*, 1993; Jenkins *et al*, 1997; Rees *et al*, 1997; Bakalakos *et al*, 1998a; Fong *et al*, 1999; Harms *et al*, 1999; Iwatsuki *et al*, 1999; Okano *et al*, 2000	0	30	7.2
				
*Multicentre*				
Resected (R0/R1 unclear)	Hughes *et al*, 1989; Kemeny *et al*, 1999a; Minagawa *et al*, 2000	32	63	35
Radical	Steele Jr *et al*, 1995		23	
Non radical	Steele Jr *et al*, 1995		14	
				
*Two centres*				
Resected (R0/R1 unclear)	Beckurts *et al*, 1997; Bradley *et al*, 1999	14	36	
Radical	Beckurts *et al*, 1997		15	
				
Resection only	Ohlsson *et al*, 1998; Harms *et al*, 1999; Iwatsuki *et al*, 1999; Kemeny *et al*, 1999a; Ambiru *et al*, 1999b	20	33.6	30
Resection+additional therapy	Steele Jr *et al*, 1995; Jamison *et al*, 1997; Ohlsson *et al*, 1998; Riesener *et al*, 1998; Bakalakos *et al*, 1998a; Bradley *et al*, 1999; Fong *et al*, 1999; Harmon *et al*, 1999; Harms *et al*, 1999; Iwatsuki *et al*, 1999; Kemeny *et al*, 1999a; Ambiru *et al*, 1999b; Kemeny *et al*, 1999c; Bolton and Fuhrman, 2000	9	63	35.5
Resection+use of additional therapy unclear	Hughes *et al*, 1989; Savage and Malt, 1992; Sugihara *et al*, 1993; Doci *et al*, 1995; Fuhrman *et al*, 1995; Scheele *et al*, 1996; Beckurts *et al*, 1997; Jenkins *et al*, 1997; Rees *et al*, 1997; Minagawa *et al*, 2000; Okano *et al*, 2000; Moroz *et al*, 2002	14	58	32.5

**Table 4:** Sites of recurrence

	**Patients**	**Liver (%)**	**Extrahepatic (%)**	**Liver+extra hepatic (%)**
Ambiru *et al*, 1999a, 1999b	Resection+HAI	14 (17.9)	16 (20.5)	9 (11.5)
(*n*=174)	Resection+PVI	5 (16.7)	12 (40)	7 (23.3)
	Resection only	19 (28.8)	20 (30.3)	12 (18.2)
	Total (R0 resection)	38 (21.8)	48 (27.6)	28 (16.1)
				
Bradley *et al*, 1999 (*n*=134)	Resection R0/R1 unclear	36 (26.9)	27 (20.1)	21 (15.7)
				
Doci *et al*, 1995 (*n*=224)	R0 resection	73 (32.6)	57 (25.4)	25 (11.2)
				
Donato *et al*, 1994	Resection only	21 (33.9)	5 (8.1)	
(*n*=102)	Resection+chemo	15 (37.5)	7 (17.5)	
	Total (R0 resection)	36 (35.3)	12 (11.8)	
				
Kemeny *et al*, 1999a, 1999b, 1999c	Resection only			24 (53.3)
(*n*=77)	Resection+chemo			8 (25.0)
	Total (R0/R1 unclear)			32 (41.6)^a^
				
Minagawa *et al*, 2000 (*n*=234)	R0/R1 unclear		73 (31.2)	98 (41.7)^a^
				
Ohlsson *et al*, 1998 (*n*=111)	R0/R1 unclear	19 (17.1)	19 (17.1)	43 (38.7)
				
Okano *et al*, 2000 (*n*=152)	R0/R1 unclear	38 (25.0)	26 (17.1)	6 (3.9)
				
Rees *et al*, 1997 (*n*=89)	R0 resection	25 (28.1)	27 (30.3)	9 (10.1)
				
Riesener *et al*, 1998	R0 resection	18 (30.5)	13 (22.0)	15 (25.4)
(*n*=109)	R0 resection+chemo	10 (20.0)	9 (18.0)	12 (24.0)
	Total	28 (25.7)	22 (20.2)	27 (24.8)
				
MSKCC, 1999 (*n*=156)	Resection+combined therapy	7 (9.5)	35 (47.3)	
	Resection+monotherapy	30 (36.6)	44 (51.8)	
	Total (R0/R1 unclear)	37 (23.7)	79 (50.6)	
				
Steele *et al*, 1995 (*n*=150)	All undergoing surgery	28 (18.7)	11 (7.3)	4 (2.7)
				
Sugihara *et al*, 1993 (*n*=109)	R0 resection	34 (31.2)	30 (27.5)	
				
Total *n*=1821		392 (21.5)	431 (23.7)	293 (16.1)

a Proportion of patients with recurrence in the liver only not specified.

**Table 5:** Prognostic factors by multivariate analysis

	**Significant (improved prognosis)**	**Nonsignificant**
Age	—	Hohenberger *et al*, 1994; Moroz *et al*, 2002
Female	Donato *et al*, 1994	Moroz *et al*, 2002
Male	Hohenberger *et al*, 1994	
		
*Primary tumour*		
Site of primary tumour	—	Sugihara *et al*, 1993; Hohenberger *et al*, 1994; Rees *et al*, 1997; Kemeny *et al*, 1999c; Moroz *et al*, 2002
Low grade of primary tumour	Scheele *et al*, 1996	Sugihara *et al*, 1993; Hohenberger *et al*, 1994; Beckurts *et al*, 1997; Rees *et al*, 1997; Harms *et al*, 1999
No regional lymph node metastases of primary tumour (Duke's stage A or B)	Doci *et al*, 1995; Fong *et al*, 1999; Minagawa *et al*, 2000	Sugihara *et al*, 1993; Hohenberger *et al*, 1994; Beckurts *et al*, 1997; Rees *et al*, 1997; Harms *et al*, 1999
		
*Liver tumour*		
Metachronous	Sugihara *et al*, 1993; Donato *et al*, 1994; Beckurts *et al*, 1997; Harms *et al*, 1999	Hohenberger *et al*, 1994; Rees *et al*, 1997; Okano *et al*, 2000
Time interval from primary (months)	Hohenberger *et al*, 1994	
	>12 (Fong *et al*, 1999)	0–3 *vs* ⩾4 (Minagawa *et al*, 2000)
	>30 (Iwatsuki *et al*, 1999)	
Size of liver metastases (cm)	Bolton and Fuhrman, 2000	
	<8 cm (Rees *et al*, 1997)	⩽2 *vs* 2.1–5. *vs* ⩾5 cm (Sugihara *et al*, 1993)
	<5 cm (Scheele *et al*, 1996; Fong *et al*, 1999; Okano *et al*, 2000)	<5 *vs* ⩾5 cm (Kemeny *et al*, 1999c)
		
		
Number of liver tumours	<8 (Moroz *et al*, 2002)	Harms *et al*, 1999; Bolton and Fuhrman, 2000
	1 (Fong *et al*, 1999; Minagawa *et al*, 2000)	1 *vs* 2–4 *vs* ⩾5 (Sugihara *et al*, 1993)
	<3 (Iwatsuki *et al*, 1999; Okano *et al*, 2000)	1 *vs* 2–3 *vs* >3 (Hohenberger *et al*, 1994; Doci *et al*, 1995; Rees *et al*, 1997)
		1 *vs* >1 (Beckurts *et al*, 1997)
		⩽7 *vs* >7 (Moroz *et al*, 2002)
		
Absence of satellite metastases	Scheele *et al*, 1996	—
Percent hepatic replacement (less involvement)	Hohenberger *et al*, 1994; Doci *et al*, 1995	—
Unilobar distribution of liver tumour	Iwatsuki *et al*, 1999	Sugihara *et al*, 1993; Hohenberger *et al*, 1994; Rees *et al*, 1997; Fong *et al*, 1999; Harms *et al*, 1999; Okano *et al*, 2000; Moroz *et al*, 2002
Fewer hepatic segments involved	Bolton and Fuhrman, 2000	—
Low/moderate grade of liver tumour	Ohlsson *et al*, 1998	—
Nodes at hepatoduodenal ligament not involved	Beckurts *et al*, 1997; Harms *et al*, 1999; Minagawa *et al*, 2000	Okano *et al*, 2000
Local invasion of hepatic disease absent	Moroz *et al*, 2002	—
Vascular invasion absent	Okano *et al*, 2000	—
Intrahepatic bile duct invasion absent	—	Okano *et al*, 2000
Extrahepatic disease absent	Ohlsson *et al*, 1998; Fong *et al*, 1999; Iwatsuki *et al*, 1999	—
Presence of fibrous pseudocapsule	Okano *et al*, 2000	—
		
Low preoperative CEA level (ng/ml)	Ohlsson *et al*, 1998	⩽5 *vs* 5.1–30 *vs* ⩾30.1 (Sugihara *et al*, 1993)
	<200 (Fong *et al*, 1999)	<50 *vs* ⩾50 (Minagawa *et al*, 2000)
		<5 *vs* >5 (Hohenberger *et al*, 1994)
		
Low postoperative CEA level (ng/ml)	(Hohenberger *et al*, 1994)	—
Increased resection margin	>5 mm (Rees *et al*, 1997)	⩾1 cm *vs* ⩽0.9 cm (Scheele *et al*, 1996)
Ro resection	Ro (Fong *et al*, 1999; Harms *et al*, 1999; Iwatsuki *et al*, 1999; Okano *et al*, 2000)	—
Type of hepatic surgery	Anatomical (Scheele *et al*, 1996)	(Sugihara *et al*, 1993; Doci *et al*, 1995; Bolton and Fuhrman, 2000)
No intraoperative blood transfusion	(Ohlsson *et al*, 1998)	—
Year of resection more recent	1985–95 *vs* 1971–84 (Ohlsson *et al*, 1998)	—
Complex *vs* simple disease	—	Bolton and Fuhrman, 2000
		
*Additional treatment*		
Chemotherapy *vs* none	—	Donato *et al*, 1994; Bolton and Fuhrman, 2000
Combined therapy *vs* monotherapy	(Kemeny *et al*, 1999c)	—

**Table A1:** Tabulation of eligible studies

							**Overall survival**			
**Study and reference(s)**	**Recruitment period**	**Study design**	**Selection of patients**	**Characteristics of patients**	**Subgroups**	**Postoperative mortality**	**Median (months)**	**1 year (%)**	**3 year (%)**	**5 year (%)**
Ambiru, 1999 Japan (Ambiru *et al*, 1999a, 1999b)	1984–1998	Retrospective Single centre	Patients undergoing radical (R0) resection only	*n*=174 Age range 21–80 years, median 63 years Men 108, women 66	(a) Total patients 174 (b) HAI chemo following resection 78 (c) PVI chemo following resection 30 (d) Resection only 66	60 days (a) 6/174 (3.4%) (b) 1/78 (1.3%) (d) 4/66 (6.1%)	(b) — (c) — (d) —	83 83 65	56 25 32	40 17 20
Bakalakos, 1998 USA (Bakalakos *et al*, 1998a, 1998b)	1978–1993	Retrospective Single centre	All patients undergoing surgery	*n*=301 Age range 25–83 years, median 61 years Men 144, women 157	(a) Total 301 patients (b) Resected group, rendered free of gross tumour 238 (i) Histopathological margins clear 94 (ii) Histopathological margins indeterminate 74 (iii) Histopathological margins involved 65 (c) Nonresected group, underwent exploration without resection because of extent or location of hepatic or EH tumour 73 (d) Incomplete resection group, had liver resections but left with gross disease in remaining liver or abdominal cavity 33	Perioperative (a) 4/301 (1.3%)	(a) 20.6 (b) 23.2 (i) 29.9 (ii) 20.5 (iii) 19.4 (c) 13 (d) 14.8	— 82 88 74 76 51 68	— 28 40 14 26 5 9	— 9 20 4 6 0 0
Beckurts, 1997 Germany (Beckurts *et al*, 1997)	1987–1994	Prospective Two centres	Patients undergoing resection	*n*=126 Age range 38–80 years, median 61 years Men 77, women 49	(a) Total patients 126 (b) Radical resection 119 (c) Nonradical resection 7	30 days 2/126 (1.6%)	(a) — (b) — (c) —	— — —	31 34 —	14 15 0
Bolton, 2000 USA (Bolton and Fuhrman, 2000)	Up to 1999	Retrospective Single centre	Patients undergoing resection	*n*=165 Age range 25–90 years, median 63 years Men and women not reported	(a) Total patients 165 (b) Simple hepatic metastases (1–3 metastatic lesions in a unilobar distribution 121 (c) Complex hepatic metastases (⩾4 distinct and separate lesions within one lobe or at least 2 distinct and separate lesions in opposite lobes) 44	Within 30 days or during same hospital stay (a) 10/165 (6.1%) (b) 6/121 (5.0%) (c) 4/44 (4.7%)	(a) —a (b) 43^a^ (c)^a^ 39	— — —	— — —	36 36 37
Bradley, 1999 USA (Bradley *et al*, 1999)	1977–1999	Retrospective Two centres	Patients undergoing resection	*n*=134 Age range not reported, mean 62 years Men 70, women 64		Perioperative 6/134 (4.5%)	—	81	50	36
Cady, 1998 USA (Cady *et al*, 1998)	Up to 1996	Retrospective Single centre	Patients undergoing resection	*n*=253 Age not reported Men and women not reported		30 days 9/253 (3.6%)	a	Disease-free survival presented for prognostic factors only		
Doci, 1995 Italy (Doci *et al*, 1995)	1980–1993	Unclear design Single centre	Patients undergoing radical (R0) resection only	*n*=224 Age range and median not reported Men and women not reported		From post surgical complications 5/224 (2.2%)	a 30	87	62	24
Donato, 1994 Italy (Donato *et al*, 1994)	1977–1990	Retrospective Multicentre	Patients undergoing radical (R0) resection only	*n*=102 Age range 35–84 years, median not reported Men 56, women 46	(a) Total patients 102 (b) Post surgical treatment: none 62 (c) Post surgical treatment: chemotherapy 40	Not reported	(a) 29 (b) 25 (c) 35	88 — —	36 — —	— — —
Fegiz, 1991 Italy (Fegiz *et al*, 1991)	Not reported	Retrospective Multicentre	Patients undergoing resection	*n*=212 Age range and median not reported Men and women not reported		1 month 14/212 (6.6%)		Not eligible		
Fernandez-Trigo, 1995 USA, Norway, Netherlands, Sweden, Germany, France, Italy, Spain (Fernandez-Trigo *et al*, 1995)	Not reported	Retrospective Multicentre	Patients undergoing repeat hepatic resection	*n*=170 Age range 28–84 years, Mean 58 years Men and women not reported		Not reported	34 From second resection	—	45	32
Figueras, 2001 Spain (Figueras *et al*, 2001)	1991–1998	Prospective Single centre	Patients undergoing resection	*n*=150 Age range not reported, mean 60 years		30 days 7/150		Not eligible		
Fong, 1999 USA (Fong *et al*, 1995, 1997, 1999; DeMatteo *et al*, 2000)	1985–1998	Retrospective Single centre	Patients undergoing resection	*n*=1001 Age range 27–87 years, median 61 years Men 581, women 420	(a) Total patients 1001 (b) Radical resection 895 (c) Nonradical resection 106	30 days 28/1001 (2.8%)	(a) 42 (b) 53 (c) 23	89 — —	57 — —	37 37 20
Fuhrman, 1995 USA (Fuhrman *et al*, 1995)	1988–1992	Retrospective Single centre	All patients undergoing surgery	*n*=151 Age range not reported, median 58.2 years Men 69, women 82	(a) Satisfied pre-op criteria 151 (b) Radical resection 107 (c) Excluded from resection 44	30 days a) 3/107 (2.8%)	(a) — (b) — (c) —	80 90 58	40 55 5	31 44 0
Harmon, 1999 USA (Harmon *et al*, 1999)	1978–1998	Retrospective Single centre	Patients undergoing resection	*n*=110 Age range 41–90 years, mean 63 years Men 65, women 45		30 days 5/110 (4.5%)	42	—	—	46
Harms, 1999 Germany (Harms *et al*, 1999)	1987–1998	Retrospective Single centre	All patients presenting with colorectal liver metastases	*n*=449 Age range 25–87 years, median 61.3years Men 253, women 196	(a) Total 449 patients (b) Resection 245 (i) Resection only 86 (ii) Resection+regional chemo 54 (iii) Resection+systemic chemo 105 (iv) Radical resection 225 (v) Nonradical resection 20 (c) No resection 204 (i) No treatment 50	30 day b) 4/245 (1.6%)	(a) 25 (b) 35 (i) — (ii) — (iii) — (iv) 64 (v) 25 (c) 16 (i) 11	78 92 91 82 95 98 70 66 —	25 38 35 35 45 45 18 15 —	15 30 30 17 35 30 11 2 —
Hohenberger, 1994 Germany (Hohenberger *et al*, 1990, 1994)	1981–1991	Prospective Single centre	Patients undergoing radical (R0) resection only	*n*=141 Age range 30–79 years, median 59 years Men 99, women 67		Post op 6/166 (3.6%)	a 30	—	—	
Hughes, 1989 USA, Germany, England (Hughes *et al*, 1988, 1989; Leslie *et al*, 1995)	1948–1985	Retrospective Multicentre	Patients undergoing resection	*n*=862 Age range and median not reported Men 418, women 327	(a) Metastatic disease isolated to liver 800 (b) Metastatic disease in liver+hepatic/coeliac nodes 25 (c) Simultaneous resection of extra hepatic metastases 37	Not reported	(a)^a^ (b)^a^ (c)^a^			32 4 20
Iwatsuki, 1999 USA (Gayowski *et al*, 1994; Iwatsuki *et al*, 1999)	1981–1996	Retrospective Single centre	Patients undergoing resection	*n*=305 Age range 26–82 years, mean 60 years Men 178, women 127	(a) All patients undergoing resection (b) Resection margin >1 cm (c) Resection margin ⩽1 cm (d) Resection margin involved	30 days 0/305 (0%) (3 deaths within 90 days)	(a) — (b) — (c) (d)	94 —	47 54.2 48.4 20.9	32.3 38.4 31.4 8.4
Jamison, 1997 USA (Rosen *et al*, 1992; Jamison *et al*, 1996, 1997)	1960–1987	Retrospective Single centre	Patients undergoing resection	*n*=280 Age range not reported, mean 59 years Men 173, women 107		30 days 5/280 (1.8%) (10 died within 60 days)	*n*=269 32.4	84	46	27
Jenkins, 1997 USA (Jenkins *et al*, 1997; Brand *et al*, 2000)	1975–1993	Retrospective Single centre	Patients undergoing resection	*n*=131 Age range 30–88 years, mean 62 years Men 72, women 59	(a) Total patients 131 (b) Radical resection 107 (c) Positive margins 14 (d) Extra hepatic disease 10	In-hospital mortality a) 5/131 (3.8%) 6/167 (3.6%)	(a) 33 (b) 36 (c) 21 (d) 18	— — —	42 50 11 29	25 30 0 0
Kemeny, 1999 USA (abstract) (Kemeny *et al*, 1999a)	Not reported	Prospective Multicentre	Patients undergoing resection	*n*=109 Age not reported Men and women not reported	(a) Total patients 109 (b) Resection only 56 (c) Resection only after withdrawals 45 (d) Resection+CHAI of FUDR and systemic infusion of 5FU 53 (e) Resection+CHAI after withdrawals 32	Operative death a) 2/109 (2.6%)	(b) 47.5 (c) 34.2 (d) (e)	91 90	66 72	32 63
Lorenz, 1997 Germany (Lorenz *et al*, 1997)	1982–1993	Prospective Single centre	Patients undergoing resection	*n*=110 Age range 35–85 years, median 62 years Men 53, women 57	(a) Total 110 pats (b) Radical resection 81 (c) Nonradical resection 29	60 days 6/110 (5.5%)		Not eligible		
Lorenz, 1998 Germany (Lorenz *et al*, 1998)	1991–1996	Prospective Multicentre	Patients undergoing resection	*n*=219 Age range 30–76 years, median 61 years Men 126, women 93	(a) Total patients 219 (b) Surgery only 111 (c) Surgery+HAI infusion 108	30 days a) 12/219 (5.5%) b) 3/111 (2.7%)c) 9/108 (8.3%)		Not eligible		
Minagawa, 2000 Japan (Minagawa *et al*, 1999)	1980–1997	Retrospective Multicentre	Patients undergoing resection	*n*=235 Age range 30–80 years, median 59.2 years Men 148, women 87		30 days 0/235 (0%)	37.2	92	51	38
Minton, 1990 USA (Minton and Abou-Issa, 1990)	1977–1989	Unclear design Single centre	Patients undergoing resection	*n*=239 Age range and median not reported Men and women not reported		30 days 0/239 (0%)		Not eligible		
Moroz, 2000 Australia (unpublished) (Moroz *et al*, 2002)	Not reported	Retrospective Single centre	Patients undergoing resection	*n*=123 Age range 30–87 years, median not reported Men 76, women 47	(a) 123 patients curative resection (b) 36 consecutive patients treated with selective internal radiation therapy+hepatic arterial chemotherapy. More advanced disease, not respectable	30 days a) 1/123 (0.8%)	(a) 38 (b) 16	88 64	53 19	31 6
MSKCC, 1999 USA (Kemeny *et al*, 1999b, 1999c)	Not reported	Prospective Single centre	Patients undergoing resection	*n*=156 Age range 28–79 years, median 59 years Men 91, women 65	(a) Total 156 pats (b) Combined therapy (HAI + systemic chemotherapy) 74 (c) Monotherapy (systemic chemotherapy) 82	Died before starting chemo (a) 5/156 (3.2%)	(a) — (b) 72.2 (c) 59.3	— — —	— — —	— — —
Nadig, 1997 USA (Nadig *et al*, 1997)	1987–1992	Retrospective Multicentre	Patients undergoing resection	*n*=275 Age not reported Men and women not reported		30 days 11/275 (4%)		Not eligible		
Nordlinger, 1992 France (Nordlinger *et al*, 1992)	1959–1991	Retrospective Multicentre	Patients undergoing resection	*n*=1818 Age range not reported, mean 60 years Men 1041, women 777		30 days 43/1818 (2.4%)		Not eligible		
Ohlsson, 1998 Sweden (Ohlsson *et al*, 1998)	1971–1995	Prospective Single centre	Patients undergoing resection	*n*=111 Age not reported Men 60, women 51	(a) Total patients 111 (b) Operative mortality excluded 107 (c) 1971–1984 68 (d) 1985–1995 43	30 days (a) 4/111 (3.6%) (c) 4/68 (5.9%) d) 0/43 (0%)	(a) 25.2 (b)^a^ — (c)^a^ 21.6 (d)^a^ 40.8	— — — —	37 — — —	25 — 19 35
Okano, 2000 Japan (Okano *et al*, 2000)	1992–1996	Retrospective Single centre	Patients undergoing resection	*n*=152 Age range 35–87 years, mean 59.5 years Men 104, women 48	(a) Total 152 patients (b) Thick fibrous pseudocapsule (10 or more bundles) 47 (c) Thin fibrous pseudocapsule (several layers of collagen bundles in histologic sections) 46 (d) No fibrous pseudo capsule 59 (e) Radical resection 121 (f) R1 resection 21 (g) R2 resection 10	Within 1 month after surgery a) 0/152 (0%)	(a) — (b) — (c) — (d) — (e) (f) (g)	91 100 93 86 96 71 80	65 88 71 41 71 44 38	58 88 64 31 67 30 25
Rees, 1997 UK (Rees *et al*, 1997)	1986–1996	Prospective Single centre	Patients undergoing resection	*n*=107 Age not reported Men and women not reported	(a) Total group 107 (b) Radical resection 89 (c) Nonradical resection 18	30 days (a) 1/107 (0.9%)	(a) — (b) — (c) —	87 94 56	47 56 11	30 37 6
Riesener, 1998 Germany (Riesener *et al*, 1998)	1986–1995	Retrospective Single centre	Patients undergoing radical (R0) resection only	*N*=109 Age range 23–83 years, mean 62 years Men 64, Women 45	(a) Total patients 109 (b) Resection only 59 (c) Resection+ chemotherapy 50	In-hospital a) 5/109 (4.6%)	(b) — (c) —	— —	— —	24 21
Savage, 1992 USA (Savage and Malt, 1992)	1962–1988	Retrospective Single centre	Patients undergoing resection	*n*=104 Age range 28–79 years, mean 60.1 years Men 53, women 51	(a) Total patients 104 (b) Radical resection 76 (c) Nonradical resection 28	Not reported	(a) 25 (b) 30 (c) 21	76 80 66	36 42 17	18 23 0
Scheele, 1996 Germany (Scheele *et al*, 1990, 1991, 1995, 1996; Stangl *et al*, 1994)	1960–1993	Prospective Single Centre	All patients presenting with colorectal liver metastases	*n*=1751 Age range 26–91 years, median 59 years Men 258, women 215	(a) Total patients 1715 (b) Total resections 498 (c) Palliative debulking 35 (d) Curative intent 463 (e) Radical resection 394 (i) Radical resection excluding operative mortality 376 (f) Non radical resection 69 (g) Unresectable, not resected 1145 (h) Resectable, not resected 102	30 days (b) 25/498 (5.0%) (c) 3/35 (8.6%) (e) 18/394 (4.6%) (f) 4/69 (5.8%)	(c)^a^ 16 (d) — (e) — (i)^a^ 41.3 (f)^a^ 14.8 (g) 7.4 (h)^a^ 16.3	— — — — — — —	— 46 52 55 — — —	0 33 38 39 — 0 5
Steele *et al*, 1995 USA (Steele Jr *et al*, 1991, 1995)	1984–1988	Prospective Multicentre	All patients undergoing surgery	*n*=150 Age range and median not reported Men 92, women 58	(a) Total patients =150 (b) Radical resection =69 (c) Nonradical resection=18 (d) Laparotomy only=63	30 days (a) 4/150 (2.7%)	(b) 35.7 (c) 21.2 (d) 16.5	90 70 70	50 25 12	23 14 0
Sugihara, 1993 Japan (Sugihara *et al*, 1993)	1978–1989	Unclear design Single centre	Patients undergoing radical (R0) resection only	*n*=159 Age range 34–82 years, mean 57.7 years Men 62, women 45	(a) Total patients =159 (b) Radical resection =109 (c) Positive resection margins=17 (d) Extra hepatic metastases=19	Postop period 2/49 (4.1%) with hepatic surgery only	(b)^a^ — (c) — (d) —	90 70 70	57.2 17 0	47.9 12 0
Taylor, 1997 Canada (Taylor *et al*, 1997)	1977–1993	Unclear design Single centre	Patients undergoing resection	*n*=123 Age range 30–87 years, mean 58.1 years Men 67, women 56		30 days 0/123 (0%)		Not eligible		
van Ooijen, 1992 The Netherlands (van Ooijen *et al*, 1992)	1979–1989	Retrospective Multicentre	Patients undergoing resection	*n*=118 Age range 28–83 years, mean 57 years Men 71, women 47		30 day 6/118 (5.1%) 1–4 months another 3 died from late complications		Not eligible		

a Operative mortality excluded.

**Table A2:** Results of quality assessment in 39 studies

	**No. of studies with response (%)**		
**Item**	**Yes**	**No**	**Unclear**
Is the hypothesis/aim/objective of the study clearly described?	37 (94.9)	2 (5.1)	NA
Are the characteristics of patients included in the study clearly described?	27 (69.2)	12 (30.8)	NA
Is the age range and mean/median of patients reported?	27 (69.2)	12 (30.8)	NA
Are the numbers of men and women reported?	30 (76.9)	9 (23.1)	NA
Are the distributions of the principal confounders clearly described?	27 (69.2)	12 (30.8)	NA
Were the subjects asked to participate in the study representative of the entire population from which they were recruited?	3 (7.7)	33 (84.6)	3 (7.7)
Were the staff, places and facilities where the patients were treated representative of the treatment the majority of patients receive?	11 (28.2)	3 (7.7)	25 (64.1)
Were data collected prospectively?	11 (28.2)	25 (64.1)	3 (7.7)
Are the main outcomes to be measured clearly described in the Introduction or Methods section?	34 (87.2)	5 (12.8)	n/a
*Were the main outcome measures used clearly defined and accurate?*			
Operative mortality	26 (66.7)	13 (33.3)	NA
Survival or recurrence	10 (25.6)	29 (74.4)	NA
Are the main findings of the study clearly described?	13 (33.3)	26 (66.7)	NA
Is the length of follow-up reported (median/mean and range/standard deviation)?	27 (69.2)	12 (30.8)	NA
Has the number of patients lost to follow up been reported and included in the appropriate analysis?	17 (43.6)	22 (56.4)	NA
Was operative/postoperative mortality included in the survival analysis?	26 (66.7)	8 (20.5)	5 (12.8)
Was surgery the only intervention received by patients in this study?	12 (30.8)	10 (25.6)	17 (43.6)

## Appendix A

Tabulation of eligible studies is illustrated in Table A1.

## Appendix B

Results of quality assessment in 39 studies are shown in Table A2.
